# Plasma Methionine and Clinical Severity in Nitrous Oxide Consumption

**DOI:** 10.3390/toxics11010012

**Published:** 2022-12-23

**Authors:** Emeline Gernez, Sylvie Deheul, Céline Tard, Marie Joncquel, Claire Douillard, Guillaume Grzych

**Affiliations:** 1 CHU de Lille, Service Hormonologie Métabolisme Nutrition Oncologie, F-59000 Lille, France; 2 CHU de Lille, Centre d’Addictovigilance des Hauts-de-France, Service de Pharmacologie, F-59000 Lille, France; 3 CHU de Lille, Clinique de Neurologie, F-59000 Lille, France; 4 CHU de Lille, Centre de Référence des Maladies Héréditaires du Métabolisme, F-59000 Lille, France

**Keywords:** nitrous oxide, methionine, amino acids, cobalamin, neurology

## Abstract

In the last few years, there has been an increase in the recreational use of nitrous oxide (N_2_O), which can lead to neurological symptoms such as sensory or motor disorders. The literature links these symptoms to a functional inactivation of vitamin B12 by oxidation of its cobalt ion, which prevents the vitamin B12 from acting as a cofactor for methionine synthase. Thus, demyelination related to methionine deficiency could be responsible for the neurological disorders associated with N_2_O consumption, including the combined sclerosis of the spinal cord. We aimed to study the correlation between the plasma methionine levels and clinical severity observed in N_2_O users. We retrospectively collected clinical and biological data from 93 patients who chronically consumed N_2_O. The patients were divided into four groups based of the severity of their clinical symptoms (based on their Peripheral Neuropathy Disability (PND) score). The plasma amino acids measurement, including methionine, were performed systematically by liquid chromatography coupled with mass spectrometry. Plasma methionine is significantly correlated with the clinical severity (Spearman coefficient: −0.42; *p*-value < 10^−5^), however, the average methionine level in the four groups is within the physiological values (N: 16–23 µmol/L). There is a significant inverse correlation between plasma methionine and homocysteine (Spearman coefficient: −0.57; *p*-value < 10^−9^), which confirms the action of nitrous oxide on the methionine synthase. A decrease in plasma methionine cannot be imputed as the only mechanism involved in the pathophysiology of the neurological disorders in nitrous oxide consumption. In addition, there are few therapeutic indications for the use of methionine. Thus, we should be careful concerning the potential use of methionine in nitrous oxide consumption. As a consequence, other pathophysiological mechanisms probably need to be identified in order to find potential therapeutic targets.

## 1. Introduction

Nitrous oxide (N_2_O) was initially used in medicine for its analgesic action and in industry as a propellant or an oxidant. In the last few few years, there has been an increase in the recreational consumption of N_2_O by inhalation in the form of cartridges or cylinders via balloons which allow the gas to be heated. However, chronic N_2_O consumption has dramatically increased in Europe [1], and it can lead to neurological symptoms which can be sensory disorders such as paresthesia or motor disorders such as walking disorders [2]. Although the neurological symptoms can be more severe with the development of a disabling motor handicap or combined subacute degeneration of the spinal cord, psychological disorders and thrombotic events have been reported [1,2,3].

The literature links neurological toxicity to a functional inactivation of vitamin B12 by oxidation of its cobalt ion [4,5]. This oxidation prevents the vitamin B12 from acting as a cofactor for the methionine synthase [4], which transform homocysteine into methionine. Vitamin B12 is also the cofactor of methylmalonyl-CoA mutase, but the impact of N_2_O on this enzyme remains to be discussed [5]. The methionine is involved in the formation of myelin. Thus, demyelination related to methionine deficiency could be responsible for the neurological disorders associated with N_2_O consumption, including the combined sclerosis of the spinal cord. Indeed, a study on fruit bats exposed to N_2_O for several weeks showed that bats supplemented with methionine showed a reduce number of neurological disorders after their exposure, which is contrary to the other groups of bats (without methionine supplementation) [6]. Thus, this study showed the protective effect of methionine on the development of neurological disorders. Methionine supplementation could therefore help in the recovery of patients with an associated deficiency. However, no data in the literature have shown a strong link between the clinical severity of nitrous oxide intoxication and the severity of the associated clinical signs.

Hence, here, we aimed to study the correlation between the plasma methionine levels and clinical severity observed in N_2_O users. Indeed, the existence of a correlation between methionine and clinical severity could explain the pathophysiology of neurological disorders during nitrous oxide intoxication, and it could also be a therapeutic option to cure the neurological symptoms in a context of nitrous oxide consumption.

## 2. Materials and Methods

### 2.1. Patients Selection and Data Collection

We retrospectively collected clinical and biological data from patients who declared recent, chronic N_2_O consumption (less than two weeks prior). The patients who consumed N_2_O who were treated in Lille University Hospital between 2020 and 2022 were included in the study (declaration number: DEC21-356). In line with the regulations set out by the French National Data Protection Commission and international recommendations [7], written informed consent was not required for this non-interventional retrospective study. All of the patients underwent both clinical and biological examinations, notably, measurements of their plasma amino acid levels as part of their routine care. Therefore, no additional tubes were collected for this study. The clinical evaluation included the Peripheral Neuropathy Disability score which was evaluated by a neurologist. The collected data (extracted from Lille University Hospital database) were clinical symptoms related to N_2_O consumption and the biological results obtained at the day of consultation or at the first day of hospitalization.

### 2.2. Rationale of Study Group Composition

To unequivocally study the relation between the plasma methionine level and clinical severity, the following approach was used. The patients were divided into 4 groups based on the severity of their clinical symptoms (based on Peripheral Neuropathy Disability (PND score)): level 0: no symptoms, level 1: patients with distal sensory disorders without gait disorders, level 2: patients with walking disorders, but they could walk without help, and level 3: patients with walking disorders who needed help or bedridden patients.

### 2.3. Amino Acids Measurement

The plasma amino acids measurements, including methionine, were performed systematically. The blood samples were collected in heparin tubes. The plasma was aliquoted and then frozen and stored at −20 °C until the analysis. The amino acids measurement was performed as described previously [8]. Briefly, proteins are precipitated by sulfosalicylic acid, and the internal standard is added. The amino acids were measured by high performance liquid chromatography on UFLC XR (Shimadzu, Kyoto, Japan) associated with tandem mass spectrometry using MRM on API 3200 Q TRAP (AB SCIEX, Framingham, MA, USA).

### 2.4. Homocysteine Measurement

A homocysteine measurement was performed systematically. The blood samples were collected in EDTA tubes. The plasma was aliquoted and then frozen and stored at −20 °C until the analysis. Homocysteine measurement was performed as described previously [9]. Briefly, dithiothreitol was used to release homocysteine from plasma proteins. Then, the proteins were precipitated by acetonitrile in acidic environment, and the internal standard, homocysteine d4, was added. Homocysteine was measured by high performance liquid chromatography on UFLC XR (Shimadzu, Kyoto, Japan) associated with tandem mass spectrometry using MRM on API 3200 Q TRAP (AB SCIEX, Framingham, MA, USA). A plasma homocysteine increase was defined as >15 µmol/L.

### 2.5. Statistical Analysis

The values are expressed as mean ± SD, mean ± SEM, or median ± IQR, as indicated in the figure legends. Statistical differences in the clinical and biological parameters were assessed using the Mann–Whitney U or Student t test for continuous variables. Values of *p* < 0.05 were considered to be statistically significant.

## 3. Results

### 3.1. Patients Characteristics

The clinical and biological data of the patients are showed in Table 1. Overall, ninety-three patients were divided over the four groups of neurological severity as follows: level 0: forty-four patients (five women and thirty-nine men), level 1: sixteen patients (three women and thirteen men), level 2: twenty-seven patients (eight women and nineteen men), and level 3: six patients (two women and four men). Thus, we notice a male predominance in all of the groups, and globally, a young age, with a mean age ranging from 20 to 26 years depending on the groups.

### 3.2. Decrease in Plasma Methionine Is Associated to Clinical Severity in Case of N_2_O Consumption

**Figure 1:**
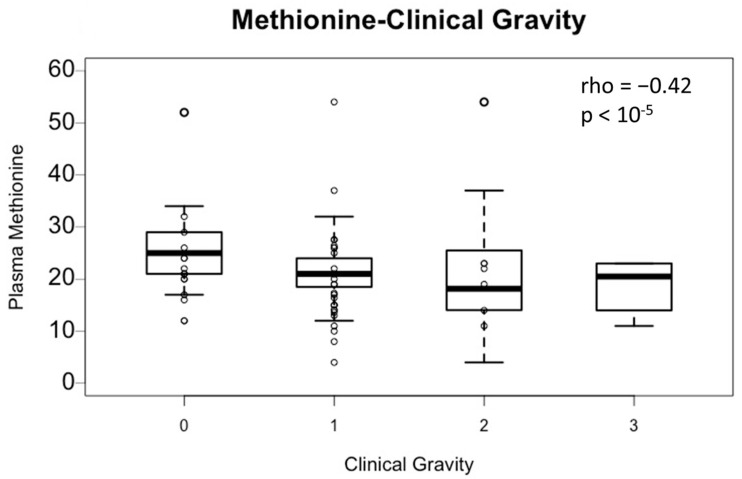
Plasma Methionine according to the clinical severity of N2O consumption. Groups are based on the severity of their clinical symptoms (based on Peripheral Neuropathy Disability (PND) score: level 0: no symptoms; level 1: paresthesia or PND I; level 2: gait disorders or PND II; level 3: thrombosis or combined subacute degeneration of spinal cord or PND III or IV. *p*-value and rho were obtained by non-parametric Spearman rank test. Data are expressed as median ± IQR.

The association between plasma methionine and clinical severity (levels 0, 1, 2, and 3) was assessed (Figure 1). Plasma methionine is significantly correlated with the clinical severity (Spearman coefficient: −0.42; *p*-value < 10^−5^). Thus, plasma methionine decreases as the clinical severity increases (group 0: mean = 25.5 ± 6.3 μmol/L, group 1: mean = 21.1 ± 5.4 μmol/L, group 2: mean = 20.0 ± 9.9 μmol/L, and group 3: mean = 18.7 ± 5.1 μmol/L). However, the average methionine level in the four groups is within physiological values (N: 16–23 µmol/L), so there is no quantitative methionine deficiency observed here.

### 3.3. Decrease in Plasma Methionine in Case of N_2_O Consumption Is Related to Homocysteine-Methionine Transformation

**Figure 2:**
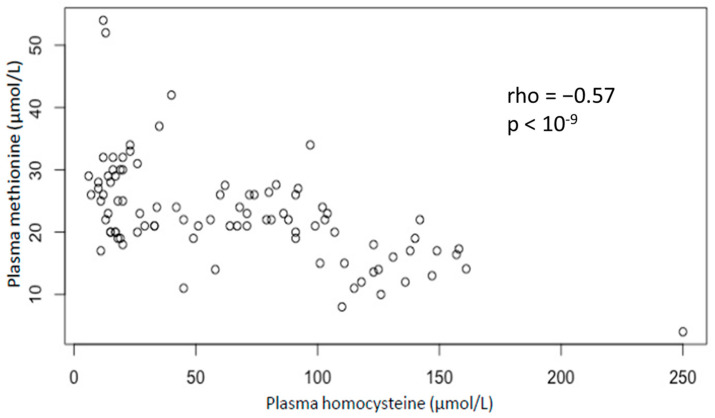
Plasma Methionine according to plasma Homocysteine. Groups are based on the severity of their clinical symptoms (based on Peripheral Neuropathy Disability (PND) score: level 0: no symptoms; level 1: paresthesia or PND I; level 2: gait disorders or PND II; level 3: thrombosis or combined subacute degeneration of spinal cord or PND III or IV. *p*-value and rho were obtained by non-parametric Spearman rank test.

**Figure 3:**
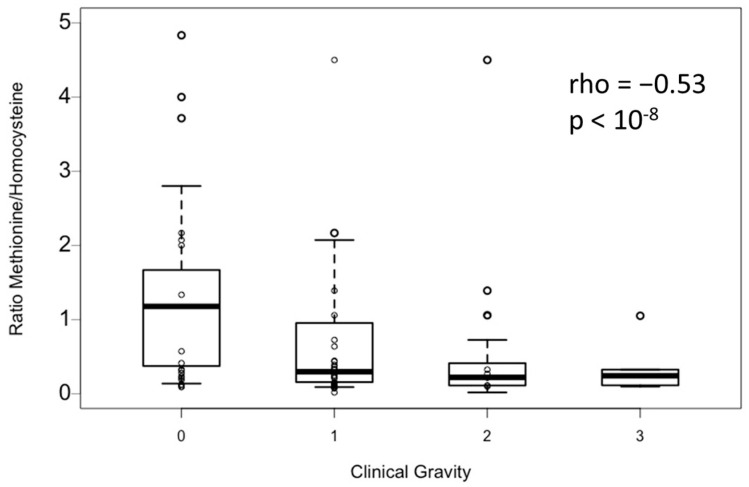
Methionine to Homocysteine ratio according to clinical severity. Groups are based on the severity of their clinical symptoms (based on Peripheral Neuropathy Disability (PND) score: level 0: no symptoms; level 1: paresthesia or PND I; level 2: gait disorders or PND II; level 3: thrombosis or combined subacute degeneration of spinal cord or PND III or IV. *p*-value and rho were obtained by non-parametric Spearman rank test. Data are expressed as median ± IQR.

The association between plasma methionine and homocysteine was assessed (Figure 2). There is a significant inverse correlation between plasma methionine and homocysteine (Spearman coefficient: −0.57; *p*-value < 10^−9^). In addition, the methionine-to-homocysteine ratio is significantly correlated with clinical severity homocysteine (Spearman coefficient: −0.53; *p*-value < 10^−8^) (Figure 3).

Hence, the decrease in plasma methionine during nitrous oxide intoxication seems to be linked to the decrease in methionine synthase activity, which confirms the action of nitrous oxide on this enzyme.

## 4. Discussion

Our study is the first to show, statistically, in a human cohort, the decrease in plasma methionine related to clinical severity in patients who chronically consume nitrous oxide. The main limitation of our study is that only a few patients are in the level 3 of clinical severity; indeed, a larger number of patients would have been more appropriate for the interpretation of the results. The decrease in plasma methionine could be used also as a blood marker, but also, on the other hand, it can be used to understand the underlying mechanisms.

Our study is consistent with the data in the literature, where the patients were administered nitrous oxide and therefore showed a rapid decrease in plasma methionine, which recovered after they stopped consuming it [10].

The same phenomenon was observed for plasma homocysteine, and it correlates perfectly with the methionine levels found. Indeed, the homocysteine/methionine ratio also correlates with clinical severity, which is consistent with a remethylation disorder and a disruption of one-carbon metabolism [11]. These changes in one-carbon metabolism have clinical consequences related to changes in many essential metabolic processes [12,13]. Hence, these metabolic changes could explain the severity of the neurological signs associated with demyelination.

However, the decrease in plasma methionine cannot be imputed as the only mechanism involved in the pathophysiology of the neurological disorders in nitrous oxide consumption. Indeed, even if our study shows an inverse correlation between plasma methionine levels and clinical severity, there is no quantitative methionine deficiency observed here. Hence, the inhibition of methionine synthase, which leads to a plasma methionine decrease, seems to play a role in the neurological severity during nitrous oxide intoxication, but it is probably not the only factor involved in the occurrence of these disorders. On the other hand, there are no reports in the literature of such severe and rapid onset clinical signs in patients with methionine or cobalamin deficiency. Additional factors, such as oxidative stress [14] or changes in transmethylation [11], must explain the pathophysiology, but this requires further studies for it to be understood.

In addition, there are few therapeutic indications for the use methionine. Indeed, it is used as a dietary supplement especially in hair loss. Additionally, methionine is currently used in a clinical trial as a treatment of Pulmonary Alveolar Proteinosis with MARS (methionyl-tRNA synthetase) mutation [15]. Thus, there is not much information concerning its toxicity. However, methionine seems to be hepatotoxic [16] and potentially neurotoxic in case of the over-dosage of it. Therefore, we should be careful concerning the potential use of methionine in nitrous oxide consumption, which needs further clinical investigation. Moreover, other pathophysiological mechanisms probably need to be identified in order to find potential therapeutic targets.

## 5. Conclusions

Our results showed that there is an association between plasma methionine and clinical severity of nitrous oxide consumption. Our results also showed that this decrease in methionine is related to a disturbance of homocysteine metabolism. However, the inhibition of the methionine synthase cannot be attributed as the only pathophysiological mechanism involved in the occurrence of neurological disorders in N_2_O consumers because there is no quantitative deficit in plasma methionine observed here. Thus, other pathophysiological mechanisms must be explored.

## Figures and Tables

**Table 1:** Characteristics of patients according to clinical severity. Groups are based on the severity of their clinical symptoms based on Peripheral Neuropathy Disability (PND) score: level 0: no symptoms, level 1: patients with distal sensory disorders without gait disorders, level 2: patients with walking disorders, but they can walk without help, and level 3: patients with walking disorders who need help or bedridden patients. Data are expressed as mean ± SD.

Clinical Severity	0	1	2	3
**N**	44	16	27	6
**Sex ratio (women/men)**	5/39	3/13	8/19	2/4
**Age (years)**	26 ± 7	25 ± 7	23 ± 3	20 ± 3
**Plasma methionine (µmol/L)**	25.5 ± 6.27	21.1 ± 5.4	20.0 ± 9.9	18.7 ± 5.1
**Plasma homocysteine (µmol/L)**	38.7 ± 35.5	74.3 ± 45.9	94.5 ± 5.5	100.8 ± 19.6

## Data Availability

The data presented in this study are available on request from the corresponding author.
